# Lithium hydroxide as a high capacity adsorbent for CO_2_ capture: experimental, modeling and DFT simulation

**DOI:** 10.1038/s41598-023-34360-z

**Published:** 2023-05-02

**Authors:** Marziyeh Ahmadi, Ahad Ghaemi, Mohammad Qasemnazhand

**Affiliations:** School of Chemical, Petroleum and Gas Engineering, Iran University of Science and Technology, Tehran, Iran

**Keywords:** Environmental sciences, Chemistry, Engineering, Materials science

## Abstract

In this work, the potential of monohydrate Lithium hydroxide (LiOH) as a high capacity adsorbent for CO_2_ capture was investigated experimentally and theoretically. The effects of operating parameters, including temperature, pressure, LiOH particle size and LiOH loading, on the CO_2_ capture in a fixed-bed reactor have been experimentally explored using response surface methodology (RSM) based on central composite design. The optimum conditions obtained by the RSM for temperature, pressure, mesh and maximum adsorption capacity were calculated as 333 K, 4.72 bar, 200 micron and 559.39 mg/g, respectively. The experiments were evaluated using isotherm, kinetic and thermodynamic modeling. Isotherm modeling showed that Hill model could deliver a perfect fit to the experimental data, based on the closeness of the R^2^-value to unity. The kinetics models showed that the process was chemical adsorption and obeyed the second order model. In addition, thermodynamic analysis results showed that the CO_2_ adsorption was spontaneous and exothermic in nature. In addition, based on the density functional theory, we investigated the chemical stability of LiOH atomic clusters and examined the effects of LiOH nanonization on the physical attraction of carbon dioxide.

## Introduction

Carbon dioxide is the main greenhouse gas, and its production is increasing every year. It is well-known that CO_2_ emissions have the major contribution in global warming, and therefore, their reduction is urgently needed^1^. The most important sources of CO_2_ emissions are power plants generating electricity from fossil fuels, cement plants, chemical and petrochemical industries, steel industry, etc.^2^. It is necessary to inexpensively eliminate or reduce carbon dioxide emissions^3^, and so, global actions against climate change must be carried out under the climate change convention and the Kyoto protocol. In 1997, various countries agreed on reducing the uncontrolled production of carbon dioxide under the Kyoto protocol targets. According to the treaty, industrialized countries pledged 2.5% reduction of their greenhouse gas emissions compared with 1990^4^. So far, different technologies have been introduced for CO_2_ capture and storage, among them physical adsorption, chemical adsorption and membrane distillation have proved to be leading choices^3,5,6^. On the other hand, CO_2_ adsorption using adsorbents, which has demonstrated to be more efficient than other methods, suffers primarily from the lack of an efficient and scalable configuration process^7–9^. Due to low cost, less corrosion, and easier recovery, this method has attracted numerous attention as a potential bid for separating carbon dioxide^10^. In the recent decades X, Y, A, ZSM-zeolites, chabazites, metal oxides, various carbons, etc. have been widely studied^11^. Among the possible candidates, solid adsorbents available for CO_2_ adsorption, such as calcium hydroxide (Ca(OH)_2_), potassium hydroxide (KOH) and sodium hydroxide (NaOH), are strong bases^6,12^, which can readily react with CO_2_ to form calcium carbonate, potassium carbonate and sodium carbonate, respectively^13,14^. The reaction of CO_2_ with hydroxides proceeds as follows^10^:

1 $$ {\text{CO}}_{2} + 2{\text{OH}}^{ - } \to {\text{CO}}_{3}^{2 - } + {\text{H}}_{2} {\text{O}} $$

The reaction between CO_2_ and hydroxides produces pure carbonate by high adsorption capacity, low thermal chemical adsorption, which is able to adsorb CO_2_ at ambient temperature and pressure^10^. Lithium hydroxide has been used for CO_2_ adsorption due to its high CO_2_ storage capacity (30 wt.%), with applications in space life support systems, space shuttle environmental control and submarine scrubbing systems^15^, (theoretically 1 kg of LiOH can absorb up to 0.91 kg of CO_2_^10^). In these applications, air or oxygen laden with CO_2_ from human or animal respiration is forced to move through a bed of lithium hydroxide granules. CO_2_ is removed and the carrier gas is returned to the environment. The reactions of water and carbon dioxide with lithium hydroxide are as follows^16^:

2 $$ 2 \, \,{\text{LiOH}}\left( {\text{s}} \right) \, + \, 2 \, \,{\text{H}}_{2} {\text{O}}\left( {\text{g}} \right) \, \to \, 2\,{\text{ LiOH}} \cdot {\text{H}}_{2} {\text{O}}\left( {\text{s}} \right),\quad \Delta {\text{H}}_{298}^{0} = - \,29.0\frac{{{\text{kcal}}}}{2}{\text{mol }}\,{\text{H}}_{2} {\text{O}} $$

3 $$ 2 \, \,{\text{LiOH}} \cdot {\text{H}}_{2} {\text{O}}\left( {\text{s}} \right) \, + {\text{ CO}}_{2} \left( {\text{g}} \right) \, \to {\text{ Li}}_{2} {\text{CO}}_{3} \left( {\text{s}} \right) \, + \, 3{\text{ H}}_{2} {\text{O}}\left( {\text{g}} \right),\quad \Delta {\text{H}}_{298}^{0} = + \,7.6\frac{{{\text{kcal}}}}{2}{\text{mol }}\,{\text{H}}_{2} {\text{O}} $$

4 $$ 2\,{\text{LiOH}}\left( {\text{s}} \right) \, + {\text{ CO}}_{2} \left( {\text{g}} \right) \, \to {\text{ Li}}_{2} {\text{CO}}_{3} \left( {\text{s}} \right) \, + {\text{ H}}_{2} {\text{O}}\left( {\text{g}} \right),\quad \Delta {\text{H}}_{298}^{0} = - 21.4\frac{{{\text{kcal}}}}{2}{\text{mol}} \,{\text{H}}_{2} {\text{O}} $$

The overall process is exothermic producing 21.4 kcal per mole CO_2_ absorbed. Reaction (4) is the classical expression and the sum effect of the intermediate reactions (2) and (3). However, some effort has been made to understand basic factors affecting the adsorption rate without considering the specific application^17^. Williams et al.^17^ studied the effect of vapor pressure and moisture percent on the adsorbent for carbon dioxide and lithium hydroxide reaction. Obviously, they proved that the pressure of water vapor in the incoming stream should be equal to or greater than CO_2_ partial pressure in order to continue the reaction. Boryta et al.^16^ investigated operating variables including the pores factors, adsorbent surface area, the partial pressure of gas, and vapor pressure of water. Wang and Bricker^18^ combined the effects of temperature and humidity on carbon dioxide absorption capacity.

Adsorption of CO_2_ onto microporous activated carbon powder was investigated in terms of isotherms, kinetic and thermodynamic^19^. It should be noted that activation of carbon in order to sensitize it to the environment should be goal-oriented. Because any type of carbon reduction does not mean effective activation in order to use it as an adsorbent^20^. Our recent research shows that the reduction of carbon structures with hydrogen reduces its interaction with the environment^21–23^. However, there are tricks to adapt activated carbon to adsorb carbon dioxide. Shi et al.^24,25^ To make carbon suitable for carbon dioxide adsorption, it was doped with nitrogen in a porous state and then activated it with potassium hydroxide or sodium hydroxide. Cui et al.^26^ showed that nitrogen doping more significant effects on enhancing the adsorption heat and selectivity. Recently, less expensive methods have been introduced to activate carbon with biomass-derived for CO_2_ uptake^27^. Odin et al. showed that the adsorption of carbon dioxide is done with spherical pheno resins, which is significantly improves by activating with potassium hydroxide^28^. Lee et al.^29^ in 2012 investigated adsorption at room temperature onto modified zeolites and activated carbon (AC) by alkali and alkaline earth metals. A fixed-bed adsorption apparatus was used to obtain more information about the effects of impregnated cations. So that, modified zeolites had greater adsorption capacities than ACs, despite their smaller surface areas, because of the electrostatic interfaces between zeolites. The intensity of the electrostatic field, and the charge density in particular, increases in the sequence of K^+^ < Na^+^ < Li^+^, resulting in enhanced electrostatic fields and greater CO_2_ adsorption capacities^29^. Cho et al. in 2015, modified the commercial zeolite of 13X and 5A with lithium hydroxide (LEZ-13X and LEZ-5A) to remove carbon dioxide in the indoor simulated examined. The BET levels of zeolite adsorbents after modification with lithium hydroxide are higher than unmodified zeolite types, as a result, it showed an increase in the adsorption capacity^30^. Krishnan et al.^31^ in 2015, proposed a system that made of activated carbon filter consists of a matrix board, lithium hydroxide and calcium hydroxide. The summarized adsorption of carbon dioxide with solid adsorbents and adsorbents modified with LiOH and are presented in Table 1.

**Table 1:** Review of carbon dioxide adsorption using solid adsorbents.

Researchers	Year	Adsorbent type	Gas composition	Q (mg/g)	T (K)	P (bar)
Lee et al.	2012	Zeolite	CO_2_–Air	39.5	298	1
Jribi et al.	2017	Activated carbon	CO_2_	1130	303	50.2
Cui et al.	2019	carbon	CO_2_–N_2_	225	273	1
Pal et al.	2020	Activated carbon-KOH	CO_2_–N_2_	1791	298	50.2
Uddin et al.	2020	phenol resin (KOH6-PR)	CO_2_	1690	298	57
Shi et al.	2020	HPCFs	CO_2_	36.08	298	20
Saeidi et al.	2018	NaOH	CO_2_	650	308	4
Williams and Miller	1970	LiOH	CO_2_–Air	132.9–611.3	298–363	~ 1
Boryta and Maas	1971	LiOH	CO_2_–He	28.3–51.2	297–333	1–29
Wang and Bricker	1979	LiOH	CO_2_–H_2_O	< 400	300	1
Kato et al.	2000	Li-ZSM-5	CO_2_–N_2_	0.02–0.06	300–400	1.2–5
Horn and Norfleet	2003	LiOH	CO_2_	0.756–0.808	298–323	High pressure
Bonenfant et al.	2008	Zeolite modified with LiOH	CO_2_–N_2_	62.39	303	2
Lee et al.	2012	Zeolite 13X modified by Li	CO_2_–Air	55	298	0.2–1
Lee et al.	2012	Activated carbon modified by Li	CO_2_–Air	12	298	0.2–1
Cho and et al.	2015	Zeolite 13X modified with LiOH	CO_2_–N_2_	198.4	298–573	1
Cho and et al.	2015	Zeolite 5A modified with LiOH	CO_2_–N_2_	58.52	298–573	1
Krishnanv et al.	2015	LiOH & Ca (OH)_2_ and activated carbon	CO_2,_ NOx, CO	2.4%	298	10–10.5

The low heat of reaction for the reaction between carbon dioxide and LiOH compared to other hydroxides, as well as the lower risks of keeping the lithium hydroxide adsorbent in a closed environment to adsorb carbon dioxide, greater compatibility with the environment and human living environment, the ability to adsorb in temperature and the ambient pressure is more justified than sodium hydroxide, potassium hydroxide and other physical and chemical adsorbents.

In this work, the kinetic, thermodynamic and isotherm of CO_2_ adsorption by LiOH was investigated experimentally and theoretically. In the design and statistical evaluation of experiments, response surface methodology (RSM) can be exploited for process modeling and optimization. RSM based on the central composite design was applied in order to design the experiments, build models and measure the optimum modification conditions to achieve desirable responses^32^. The main objective of this work is to explore the influence of modification parameters on the CO_2_ adsorption performance of the solid adsorbents in a fixed-bed reactor. In addition, the adsorption process of carbon dioxide by lithium hydroxide atomic cluster has been simulated. The simulations were performed based on the density functional theory. The simulation results are about the type of interaction between CO_2_ and lithium hydroxide at room temperature, and the effect of the size of grains on the adsorption of carbon dioxide gas.

## Materials and producers

### Materials

Lithium Hydroxide (LiOH) was purchased from Merck chemical Co., and purified carbon dioxide gas (99.98%) was supplied by Sabalan Gas Co. (Tehran, Iran). LiOH is a solid powder and density, melting point, and solubility of LiOH sorbent in water are 2540 kg/m^3^, 20 °C and 12.8 g/100 g, respectively.

### Characterization of adsorbents

The solids and liquids were analyzed to identify the links and chemical structure. Laboratory FTIR spectrometer system, is able to pass and adsorption spectra of liquid, solid and powder. The FTIR spectroscopy analysis was performed using a spectrometer (Perkin Elmer, Model 2000 FTIR, USA) to identify surface functional groups in LiOH. X-ray diffraction is used to identify the chemical composition and properties of crystalline crystals, ceramics, metals, alloys and synthetic materials widely used in chemical engineering.

### Adsorption setup

All CO_2_ adsorption experiments were performed by Lithium hydroxide with mesh of No70. The laboratory set up includes three parts: (1) gas infusion device, (2) CO_2_ reactor device, (3) investigation of CO_2_ pressure changed in the reactor during uptake process. The reactor length, inner radius and the internal volume were 9 cm, 3 cm and 255 cm^3^, respectively. At the beginning of the test, CO_2_ transferred from the cylinder to the reactor encasement via pressure current supervisors. As well as, the required temperature for each experimental run was provided by thermocouple linked to the reactor body and controlled via regulating the set point for the system. Changing in the temperature and pressure for 1 h of the reactor comprising solid adsorbent were analysis and control online during the process. All data were stored in separate Excel files in a reference computer with the temperature, pressure, time and the date. The pressure, temperature and amount of the adsorbent ranged between 1 and 9 bar, 298–363 K and 2.4 g, respectively. When pure CO_2_ was injected to the reactor containing solid adsorbent, adsorption process was began and CO_2_ was captured through the solid adsorbent. During the CO_2_ adsorption process, the pressure in the reactor was decreased. The CO_2_ adsorption rate as the difference between initial and final of CO_2_ pressure by the gas sensor was measured. The adsorption capacity of the adsorbent was calculated through the following equation:

5 $$ q_{e} = \frac{{\left( {P_{i} - P_{e} } \right)vM_{{{\text{CO}}_{2} }} }}{RTmz} $$

*K*_*d*_ is the distribution coefficient (cm^3^/g). The distribution coefficient was calculated through the following equation:

6 $$ k_{d} = \frac{{P_{{{\text{CO}}_{2} }}^{initial} - P_{{{\text{CO}}_{2} }}^{final} }}{{P_{{{\text{CO}}_{2} }}^{final} }} \times \frac{v}{w} $$

where *w* is the weight of adsorbent. *q*_*e*_ is the adsorption capacity (mg/g), *P*_*i*_ is the initial pressure (bar), *P*_*e*_ is the equilibrium pressure (bar), m is the dosage of adsorbent used (g) and *v* is the volume of the gas (L^-1^). The adsorption percentage of CO_2_ was calculated as follows:

7 $$ Adsorption\left( \% \right) = \frac{{P_{i} - P_{f} }}{{P_{i} }} \times 100 $$

where *P*_*i*_ and *P*_*f*_ are the initial and final pressure, respectively. Also, the correlation coefficient (R^2^), which represents the variability percentage in the dependent variable (the variance about the mean) is employed to analyze the fitting degree of isotherm and kinetic models with the experimental data (Eq. 8)^33^. Its value may vary from 0 to 1^34^.

8 $$ R^{2} = \frac{{\left( {q_{e,meas} - \overline{{q_{e,calc} }} } \right)^{2} }}{{\mathop \sum \nolimits \left( {q_{e,meas} - \overline{{q_{e,calc} }} } \right)^{2} + \left( {q_{e,meas} - q_{e,calc} } \right)^{2} }} $$

where *q*_*e,meas*_ and *q*_*e,calc*_ are the measured and calculated adsorption capacity (mg/g), respectively.

## LiOH atomic clusters

The density functional theory (DFT) was applied for analysis of carbon dioxide adsorption by LiOH atomic clusters. Previously, using the density functional theory, extensive research has been done on the interaction of carbon dioxide and lithium compounds^35–41^. The nanonization effects of LiOH salt on carbon dioxide adsorption was investigated. To investigate the effect of carbon impregnated with lithium hydroxide on carbon dioxide capturing, we performed modeling based on density functional theory (DFT). The calculations are based on the B3LYP functional^42–46^, and the electron density is modelled with the LANL2DZ basis set^47,48^. Our DFT simulations are performed using the Gaussian software package^49^.

## Response surface methodology

The effects of the three independent variables, including temperature, pressure and LiOH particle size (mesh size), on the CO_2_ adsorption capacity was explored using the central composite design. These variables, along with their respective regions of interest, were selected based on the literature and preliminary investigations^50^. Table 2 presents the range and levels of the independent numerical variables in terms of actual and coded values.

**Table 2:** Independent numerical variables and their levels (actual and coded).

Code	Independent numerical Variables	Unit	Symbol	Coded variable levels − 1 0 1 2
A	Temperature	°C	X_1_	30, 50, 70, 90
B	Pressure	bar	X_2_	3, 5, 7, 9
C	Particle size (mesh size)	µm	X_3_	200, 300, 500, 800

## Results and discussion

### Adsorbent characterization

The adsorbent FTIR spectrum is important for evaluating the presence of functional groups and desired links. The FTIR test result for dry lithium hydroxide powder was well displayed (Fig. S1 in the supplementary). At the beginning of the reaction, only solid LiOH was available. Peaks available in the FTIR test for lithium hydroxide are shown at 3568.14 cm^−1^, 1576.70 cm^−1^, 997.10 cm^−1^. Accordingly, these peaks can represent oxygen and O–H bonds. It should be noted that at 3568.14 cm^−1^, in lithium hydroxide, a larger peak is shown in comparison with Li_2_CO_3_. Peak 3568.14 cm^−1^ is related to O–H link. But in the analysis of Li_2_CO_3_ FTIR, peaks 3568.01, 2363.67, 1443.40 and 863.15 cm^−1^ are well visible, respectively, for O–H, C≡C, C=O, and C–H bonds. Due to the presence of carbon and oxygen bonds in lithium carbonate, carbon-rich peaks in FTIRs were well visible. Li peak will appear at less than 300 cm^−1^, according to the existing device, it is not possible to display peaks less than 400 cm^−1^. The only LiOH product was before the adsorption process (Fig. S2 in the supplementary). To ensure the presence of lithium hydroxide in the adsorbent, the XRD analysis is taken. Spreading peaks at 29.8, 33.36, 36.76 and 57.68 degrees can indicate lithium hydroxide in the XRD pattern^51,52^.

### Response surface methodology results

In the present work, response surface methodology (RSM) explores the relationships between input variables including temperature, pressure and LiOH particle size (mesh size) and CO_2_ adsorption capacity as a response variable. The RSM method was applied to design the experiments and investigate the optimization of the process. In the RSM, A-temperature, C-mesh size, B-pressure, AB, and C^2^ affect the response variable significantly; all these outcomes have been taken from the information given in the p-value column (Table S1 in the supplementary). The P-value model is lower than 0.001, which shows that the model terms are highly significant. P-values less than 0.05 and 0.001 indicate that the model terms are significant and highly significant, respectively. While P-values greater than 0.1 indicates that the model terms are not significant. The same way, f-value substantial range is 1–40^53,54^.

In order to industrialize the alkali metal-based sorbent, the effects of the operation conditions on CO_2_ capture performance should be determined in details (Table S2 in the supplementary). There are 8 factorial points, 6 axial points and 6 replicates at the center points, indicated by a total of 20 experiments, as calculated from N = 2^n^ + 2n + n = 20.

In addition, the experimental values for responses are in good agreement with the amounts predicted by the RSM model. The predicted values, were more close to the experimental values, due to possessing high R^2^ (Fig. S3 is in the supplementary).

**Figure 1:**
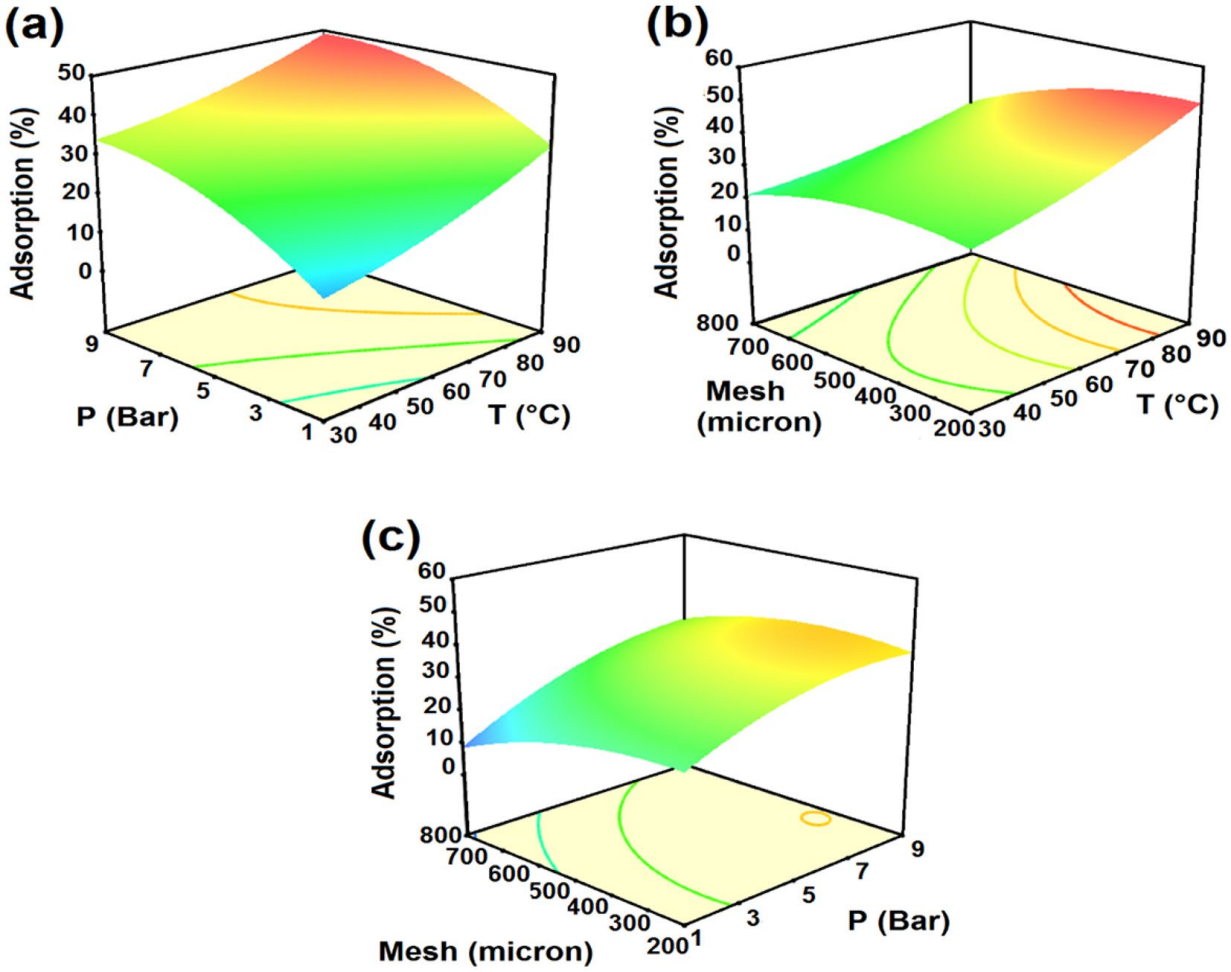
CO_2_ adsorption percentage of LiOH with (**a**) pressure and temperature, (**b**) temperature and mesh size, (**c**) pressure and mesh size.

**Figure 2:**
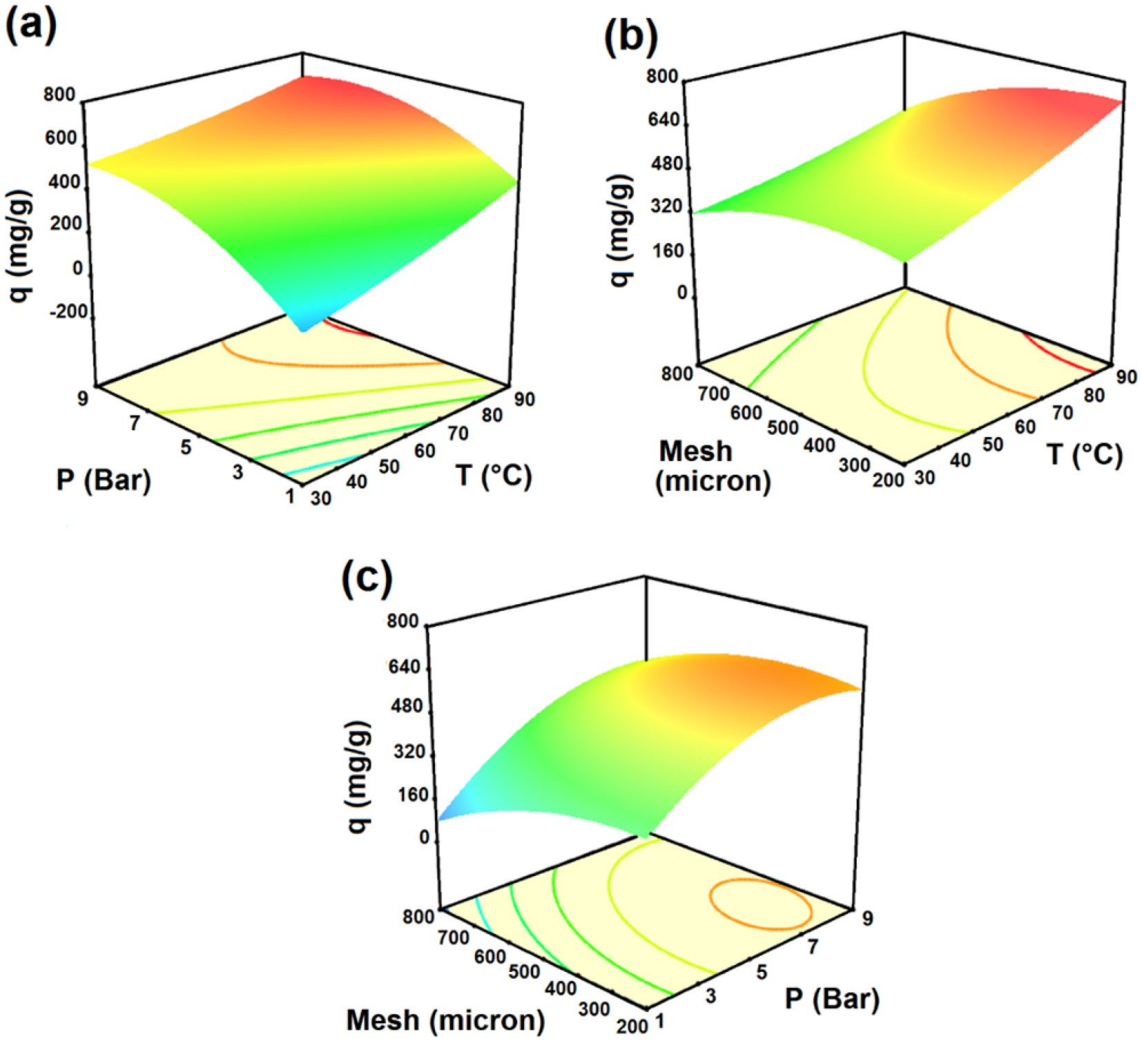
CO_2_ adsorption capacity of LiOH with (**a**) pressure and temperature, (**b**) temperature and mesh size, (**c**) pressure and mesh size.

The interaction of operating parameters in CO_2_ adsorption was obtained by RSM. Figures 1 and 2 illustrate the dimensional response surfaces, which show the effects of the significant variables. Figures 1a,b and c illustrate the dimensional response surfaces, which show the effects of the significant variables including pressure, temperature and adsorbent mesh on CO_2_ adsorption percentage. The Figures show that increasing pressure and temperature resulted in an increase in the adsorption percentage, while increasing the adsorbent mesh size led to a decrease in the adsorption percentage.

Figures 2a,b and c show the dimensional response surfaces, which show the effects of the significant variables including pressure, temperature and adsorbent mesh on CO_2_ adsorption capacity. It is clear that pressure and temperature have positive effects on CO_2_ adsorption capacity whereas increasing the mesh size of the adsorbent has negative effects on CO_2_ adsorption capacity. Surface area of the adsorbent increased by decreasing the mesh size, and consequently, adsorption resistance of the adsorbent decreased.

### Isotherm modeling

To optimize the design of CO_2_ adsorption system, it is important to determine the appropriate mechanisms and describe the thermodynamic equilibrium quantitatively. Hence, it is necessary to understand the equilibrium to predict the adsorbent behavior. Therefore, the equilibrium experimental data for CO_2_ adsorbed in the lithium hydroxide adsorbent were investigated using Langmuir isotherm, Freundlich, Dubinin-Radushkevich (D-R) and Hill. The constant values of Langmuir, K_L_ and q_m_ of the K and n constants for the Freundlich, the q_m_ and E constants for Dubinin-Radushkevich, and so on, the constants for the Hill, models at 303 K, are given in Table 3. The Langmuir equation is given by Eq. (9) below:

9 $$ q_{e} = \frac{{q_{m} K_{L} P_{{{\text{CO}}_{2} }} }}{{1 + K_{L} P_{{{\text{CO}}_{2} }} }} $$

where q_e_ is the amount of CO_2_ adsorbed at equilibrium (mg/g), q_m_ is the maximum adsorption capacity of the adsorbent (mg/g), $P_{{{\text{CO}}_{2} }}$ is the equilibrium pressure of the gas adsorbed (bar), and K_L_ is the Langmuir adsorption constant that relates to the free adsorption energy (1/bar)^55^. The Freundlich isotherm model is the earliest known relationship that presents a non-ideal and reversible adsorption process^56^. Freundlich is applicable to the multilayer adsorption, and it is based on an assumption that the adsorption energy will exponentially decrease with an extent of the adsorption process^57^. The model can be expressed by Eq. (10)^50^:

10 $$ q_{e} = k_{1} \times P_{{{\text{CO}}_{2} }}^{\frac{1}{n}} $$

where k_1_ is the distribution coefficient and n is related to a correction factor. As the k_1_ value increases, the adsorption capacity of adsorbent for a given adsorbate will also increase. The adsorption capacity or surface heterogeneity is determined from the slope value of 1/n, in which n is between 0 to 1, and by shifting the value towards zero, it becomes more heterogeneous. Contrary to the Freundlich and Langmuir model, the Dubinin–Radushkevich (D-R) isotherm can be used to describe adsorption on both homogenous and heterogeneous surfaces. The isotherm equation can be expressed by Eq. (11), as follows^58^:

11 $$ q_{e} = q_{s} \times exp\left( {K_{ad} \times \left( {R \times T \times log\left( {1 + \frac{1}{{P_{{CO_{2} }} }}} \right)^{2} } \right)} \right) $$

where R is the gas constant (8.314 J/mol K) and T is the absolute temperature. Dubinin-Radushkevich isotherm is an experimental model initially conceived for the adsorption of subcritical vapors onto micropore solids, following a pore filling mechanism, which is generally applied to express the adsorption mechanism with a Gaussian energy distribution onto a heterogeneous surface^59^.

**Table 3:** Isotherm models parameters for CO_2_ adsorption.

Model	Parameter	Value	R^2^
Langmuir	q_m_	410.28	0.9778
	K_l_	14.076	
Freundlich	K	317.20	0.9850
	N	5.435	
D-R	q_m_	403.71	0.9930
	E	9.821	
Hill	q_s_	517.997	0.9989
	N	0.474	
	K_d_	0.504	

**Figure 3:**
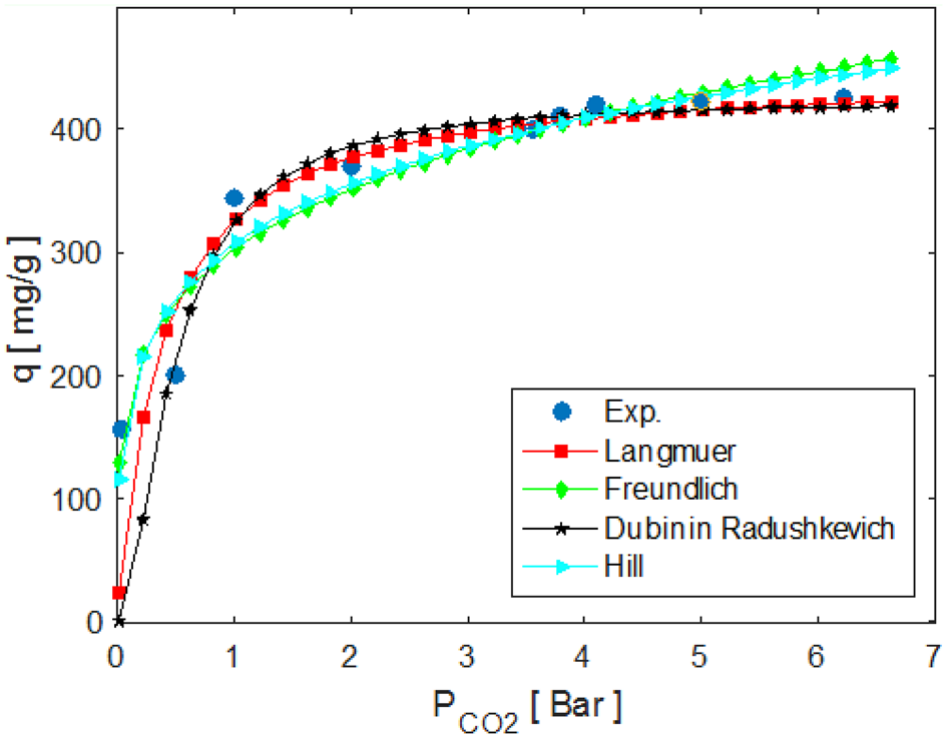
Experimental data and isotherm models for CO_2_ adsorption at 303 K.

Following the introduction of the isotherms, the Hill isotherm model, originated from the NICA model^58–60^, was postulated. The model describes binding of different species onto homogeneous substrates. The model assumes that the adsorption is a cooperative event, and the binding ability of the ligands at one site on the macromolecule are similar with other macromolecule binding sites. This Hill isotherm model is defined by Eq. (12)^59^:

12 $$ q_{e} = q_{s} \times \frac{{P_{{{\text{CO}}_{2} }}^{nH} }}{{\left( {K_{D} + P_{{{\text{CO}}_{2} }}^{nH} } \right)}} $$

where n_H_ is the constant incorporating both Langmuir and Freundlich isotherm models for representing the equilibrium adsorption data. In Fig. 3, the experimental comparison of data with Langmuir, Freundlich and D-R models is well represented at 303 K. The desirability of the adsorption process is shown by correlation coefficient (R^2^).

In a component isotherm study, determining the best-fitting model is the key analysis to mathematically describe the adsorption system. With respect to the R^2^ values, the suitability of these models in predicting the sorption behavior follows the order of Hill > D-R > Freundlich > Langmuir. Furthermore, the computed value of n_F_ < 1 implies that the CO_2_ adsorption onto the LiOH is a chemisorption process, whereas if the value is larger than 1, it suggests a physisorption process^61^. In Table 3, the value of R^2^ is in the range of 0–1, which shows that CO_2_ adsorption is desirable or not. According to R^2^, the most suitable models for predicting adsorption behavior are Hill > D-R > Freundlich > Langmuir. Perez et al. suggested that Langmuir’s model is the best one for describing the chemical reactions due to its limitation to one layer. While Freundlich primarily represents the process of physical adsorption because it allows adsorbent molecules to form a continuous layer on the adsorbent surface. The CO_2_ adsorption is shown in the range of 0–2 by a 1/n constant Freundlich. The value of 1/n < 1 shows that the CO_2_ adsorption onto the adsorbent is chemical adsorption. While for the value of 1/n > 1, the adsorption would be a physical process. The D-R isotherm provides useful information about energy parameters. E is defined as the energy of free adsorption. Accordingly, the value of E < 8 kJ/mole corresponds to a physical adsorption, the value in the range of 8 < E < 16 indicates that the adsorption is controlled by the ion exchange mechanism, and the value of E > 16 kJ/mol shows that the adsorption is due to the influence of particle penetration^62–64^ and it is a chemical adsorption.

### Kinetics modeling

**Figure 4:**
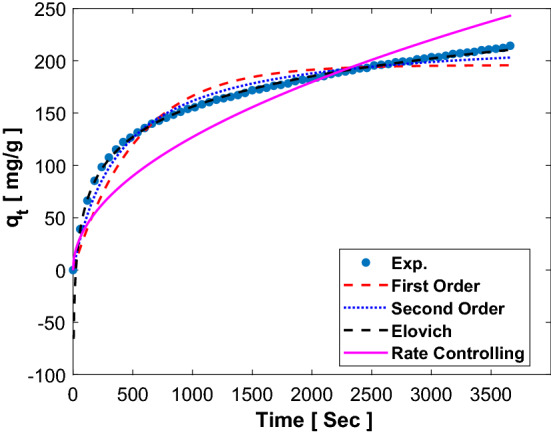
Experimental data and kinetics models for CO_2_ adsorption on LiOH.

**Figure 5:**
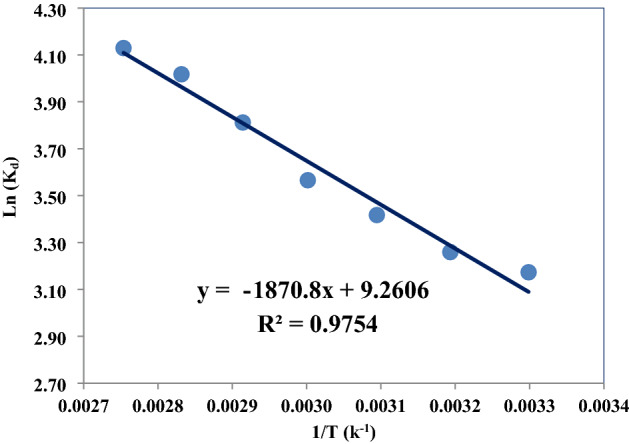
Plot of Ln k_d_ versus 1/T for adsorption of CO_2_ on LiOH.

Because of the complexity of predicting kinetic parameters, a common approach involves matching empirical data to a set of fixed models and choosing the best one. Among all the existing kinetic models presented, the first-order and second-order models are the easiest to describe the kinetics of CO_2_ adsorption (Table S3 in the supplementary). The another applicable kinetics models are Elovich, Ritchie second order and Rate control. The results of the models are presented in Fig. 4. Specifications and parameters of each model are presented separately at temperature in range of 303 to 363 K. As the results of the correlation coefficient R^2^ in the Table 4, the first-order model is weak, while the second-order models are suitable for all experimental results. In this model, the parameter R^2^ is very close to the unit value. In particular, the first-order model can indicate the reciprocal interaction between adsorbent and adsorption, which is suitable for predicting the behavior of CO_2_ adsorption in physical adsorbents, such as activated carbon and zeolite. On the contrary, the second-order model assumes that the interaction between adsorbent and adsorption is caused by a strong gas connection to the adsorbent surface, which is more suitable for chemical moieties and CO_2_-adsorption processes involving chemical interactions and chemical bonding. The CO_2_ adsorption on the lithium hydroxide is well suited for chemical adsorption. In Table 4, the value of R^2^ is in the range of 0–1, which shows that CO_2_ adsorption is desirable for second-order model. In Fig. 5, the experimental comparison of data with first order, second order, Elovich and Rate controlling models is well represented at 303 K.

**Table 4:** Kinetic models parameters for CO_2_ adsorption on LiOH at pressure of 6 bar.

Kinetic model	Parameter	Temperature (K)			
		303.15	323.15	343.15	363.15
First order	qe	195.79	172.55	244.75	280.66
	K1	0.0020	0.0044	0.0036	0.0042
	R2	0.967	0.976	0.998	0.998
Second order	qe	224.71	185.95	265.63	300.45
	K2	1 × 10^–5^	4 × 10^–5^	2 × 10^–5^	2 × 10^–5^
	R2	0.989	0.998	0.994	0.999
Elovich	Α	0.001	0.017	0.006	0.016
	Β	41.856	25.438	39.220	39.190
	R2	0.999	0.964	0.921	0.876
Rate controlling	Kid	4.016	3.768	5.276	6.075
	R2	0.963	0.843	0.804	0.746

### Adsorption thermodynamics

In the adsorption processes, thermodynamic factors including entropy and Gibbs free energy should be considered in order to determine which adsorption process will occur spontaneously. Enthalpy change (ΔH°), Gibbs free energy change (ΔG°) and entropy change (ΔS°) can be estimated using equilibrium constants changing with temperature. The distribution coefficient at constant temperature was calculated using Eq. (17).

17 $$ {\text{Ln}}K_{d} = \frac{{\Delta S^{o} }}{R} - \frac{{\Delta H^{o} }}{RT} $$

where *K*_*d*_ is the distribution coefficient (cm^3^/g), ΔS° is entropy change, ΔH° is enthalpy change, T is the absolute temperature (*K*), R is gas constant (kJ/mol K). The standard free energy values was calculated using Eq. (18).

18 $$ \Delta G^{o} = \Delta H^{o} - T\Delta S^{o} $$

The values of the enthalpy change and entropy change are calculated from the slope and intercept of the plot of Ln(*K*_*d*_) versus (1/T) as presented in Fig. 5. The values of ΔH°, ΔS° and ΔG° are listed in Table 5. It is clear that the adsorption reaction of CO_2_ on LiOH is endothermic. The free energy value for all the temperatures is negative, and the decrease in the value of ΔG° with increase in temperature shows that the reaction can be done easier at high temperatures.

**Table 5:** Thermodynamic parameters of CO_2_ adsorption on LiOH.

ΔH° (kJ mol^−1^)	ΔS° (kJ mol^−1^ K^-1^)	ΔG°(kJ mol^−1^)						
		303.15	313.15	323.15	333.15	343.15	353.15	363.15
–14.301	− 0.073	− 36.484	− 37.216	− 37.947	− 38.679	− 39.410	− 40.142	− 40.874

The experiments were carried out at 303–363 K and pressure of 6 bar of CO_2_. The values of ΔH° and ΔS° were calculated from the slopes and intercepts of linear regression of Ln k_d_ versus 1/T. The results in Table 5 indicates that the process is exothermic and the adsorption capacity of the adsorbent increases with increase in temperature. The results shows that the adsorption percentage has increased with increasing the adsorption temperature (Fig. S4 in the supplementary). The negative value of ΔH indicates that the adsorption process is exothermic and shows the chemical reaction between the gas and adsorbent. As the temperature rises, the reaction progresses and the adsorption capacity increases, resulting in irreversibility of the reaction between CO_2_-lithium hydroxide in the reactor and the production of lithium carbonate. The negative value of ΔS represents the tendency to the adsorbent material and some structural changes in the adsorbent and the absorbent. On the other hand, negative entropy indicates an increase in irregularities during adsorption and a slight change in the absorbent and adsorbent structural changes, and thus, indicating a spontaneous reaction. significant enthalpy changes indicate that the process is sensitive to temperature, and conversely, slight enthalpy changes indicate that the adsorption process is not sensitive to temperature ^65,66^.

### Effect of operational conditions on adsorption capacity

**Figure 6:**
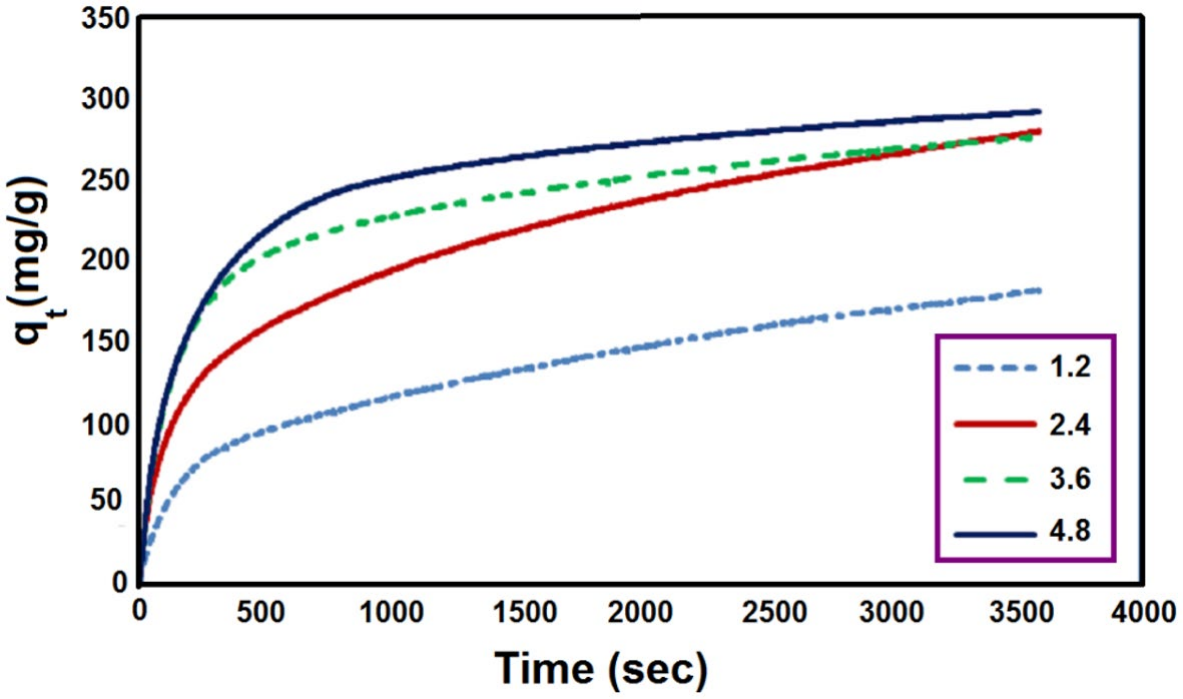
The effect of different LiOH loading on adsorption capacity CO_2_ (mg/g) at 303 K.

**Figure 7:**
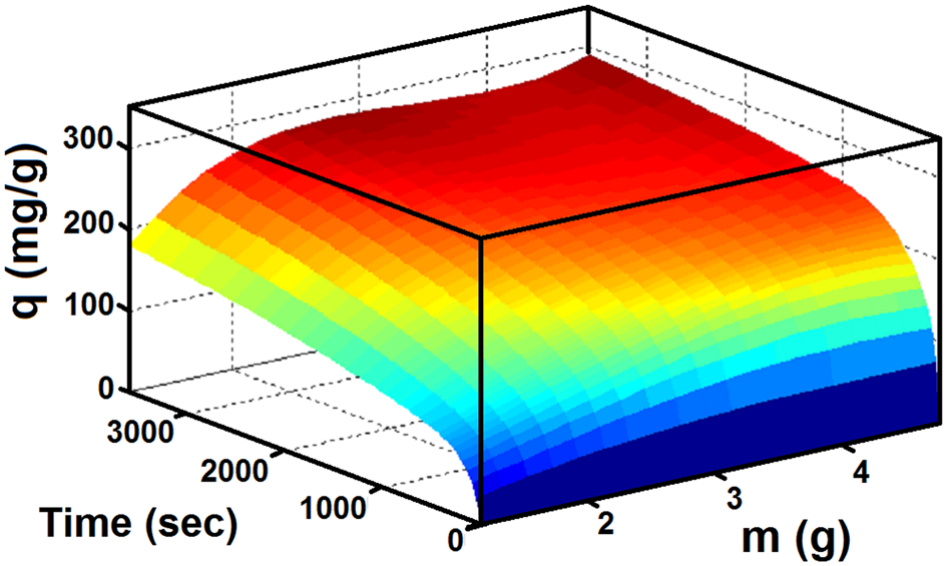
Adsorption capacity with time and different LiOH loading.

The effect of different LiOH loadings on CO_2_ adsorption capacity is illustrated in Fig. 6. It is clear that increasing the adsorbent loading results in an increase in the adsorption of CO_2_. The lowest adsorption capacity of 180.196 mg/g was exhibited by the lowest LiOH dosage of 1.2 g, and further dosage from 2.4 to 4.8 g did not substantially increase the CO_2_ adsorption capacity (277.99 and 290.127 mg/g). Due to this, the adsorption capacity of CO_2_ was determined at an optimal adsorbent of 2.4 g. Since the CO_2_ adsorption reaction with lithium hydroxide is associated with the production of water, the production of water during the reaction requires heat, and the overall reaction is a thermal one. Hence, by increasing the amount of the adsorbent, the heat required for the second reaction is provided. Similarly, Fig. 7 shows a schematic representation of increasing the amount of adsorption by increasing the amount of the adsorbent.

The results show the effect of particle size of lithium hydroxide on CO_2_ adsorption capacity. The solid lithium hydroxide was crushed using a mortar and passed through a mesh strainer. The particle size was determined in meshes of 200, 300, 500 and 800 microns. Reducing the particle size of the Lithium Hydroxide powder leads to an increase in the CO_2_ adsorption capacity (Fig. S5 in the supplementary).

**Figure 8:**
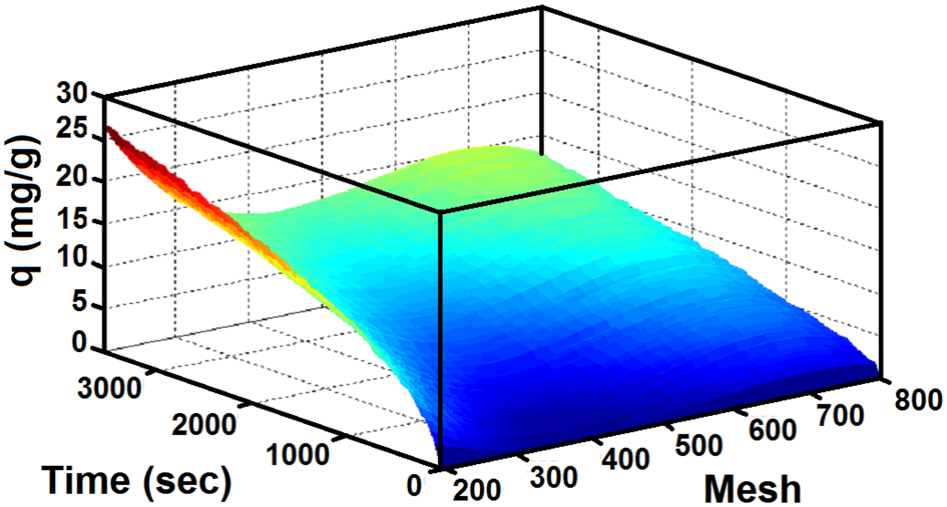
The effect of particle size on carbon dioxide adsorption capacity.

The adsorption experiments were carried out using various particle sizes of the adsorbent (diameter of the LiOH particles 200 to 800 µm) at 6 bar and 303 K. The analysis of CO_2_ adsorption at different particle sizes showed that the CO_2_ removal rate increased with a decrease in the particle diameter (Fig. 8). In this temperature and pressure, that smaller particles possess large surface areas, the required time to reach equilibrium for fine particles is less of the time required for the coarse particles. The reduction of particle diameter caused to raise the gas–solid contact surface resulting in a faster equilibrium achievement^67^.

**Figure 9:**
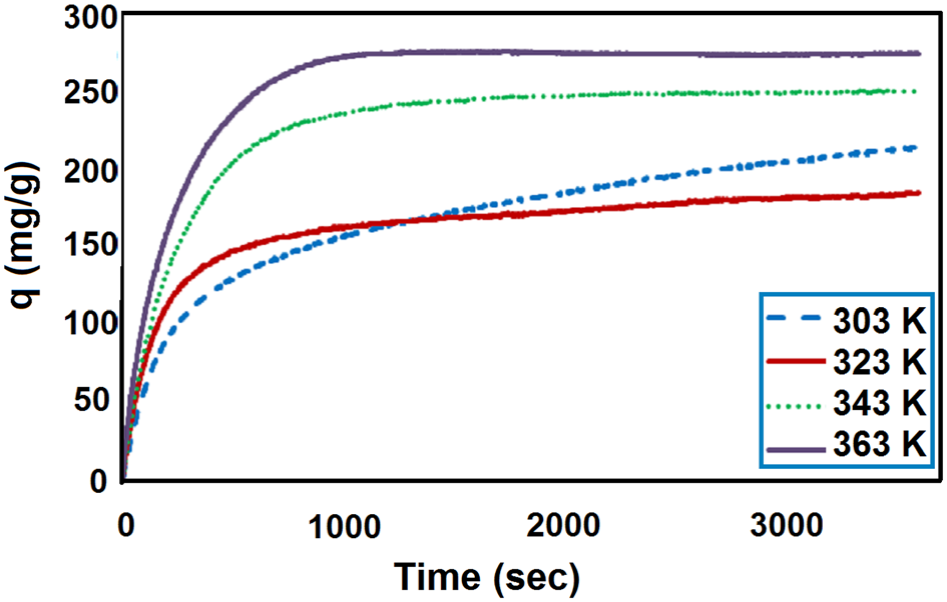
The effect of temperature on CO_2_ adsorption capacity.

**Figure 10:**
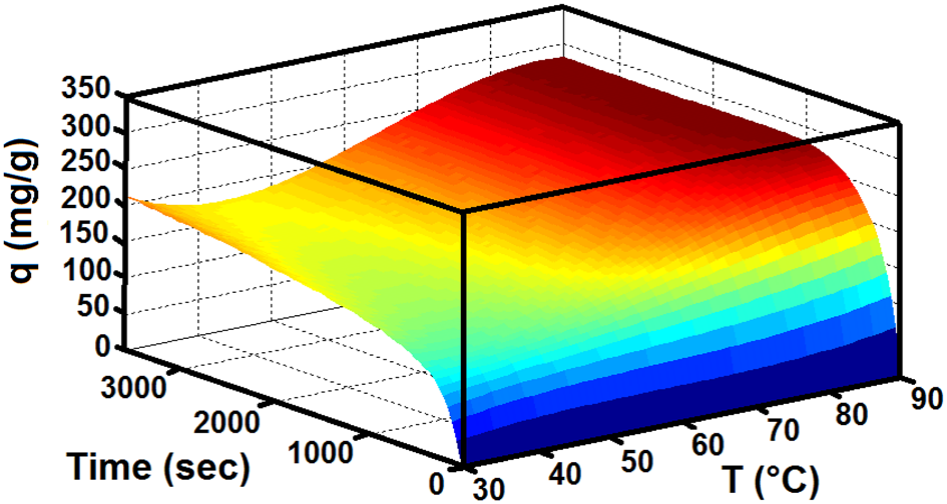
The effect of temperature with time on the CO_2_ adsorption capacity.

The effect of different adsorption temperatures, including 303, 323, 343 and 363 K, on the CO_2_ adsorption capacity is shown in Fig. 9.The amounts of adsorption at higher temperatures show that the adsorption capacity increases at higher temperatures. This trend of higher adsorption capacity with high temperatures indicate that the adsorption of CO_2_ on LiOH is chemical adsorption. The catalytic effect of water on the adsorption of CO_2_ has been proved by Miller and Piatt^69^ to have a substantial effect on the reaction, as exhibited in the Eq. (3). Boryta and Maas^16^ suggested that in order to absorb CO_2_ effectively, a reactive intermediate lithium hydroxide monohydrate, LiOH·H_2_O, should be first formed (reaction Eq. (2)). Moreover, according to the FTIR analysis, the surface of LiOH contained some –OH, which may be responsible for the CO_2_ adsorption at low temperatures. The reaction of lithium hydroxide with CO_2_ is an exothermic reaction due to the acid strength and power of lithium hydroxide. Similarly, Fig. 10 shows a schematic representation of increasing the amount of adsorption by increasing the temperature.

**Figure 11:**
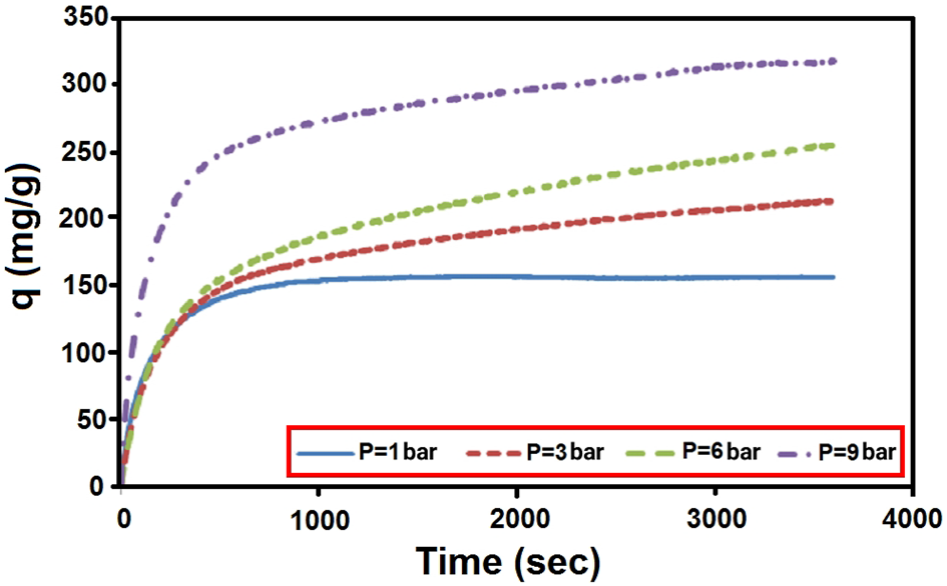
Experimental adsorption capacity of CO_2_ on LiOH at different pressure.

**Figure 12:**
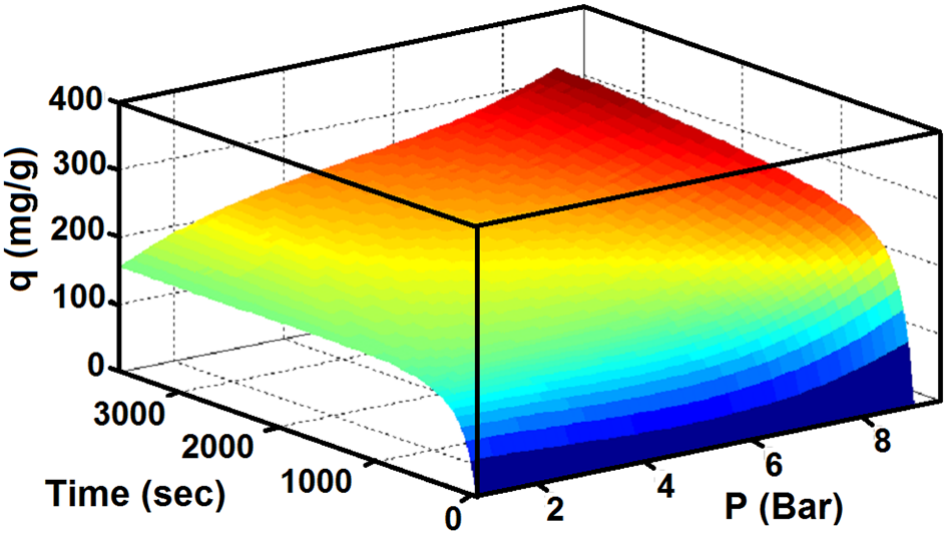
CO_2_ adsorption capacity of lithium hydroxide with time and pressure.

Pressure is one of the most important parameters in the adsorption processes. The effect of pressure and time on adsorption capacity are presented in Figs. 11 and 12 at temperature of 303 K. In Figs. 11 and 12, it is clear that the highest CO_2_ adsorption capacity was achieved at pressure of 9 bar, demonstrating that pressure had a positive effect on the adsorption capacity. This trend is on a higher upward pressure level so that at high pressures, the equilibrium is not noticeably visible and adsorption is still ongoing. This leads to an increase in adsorption capacity by increasing pressure. The equilibrium constant increases with increasing pressure, and therefore, the amount of molecules filled on the surface increases. The effect of pressure on increasing the position of molecules in adsorbent vacant sites and unreacted adsorbent portions leads to increased gas adsorption capacity. In this way, air can simulate in high pressure and low pressure environments. It is noteworthy that these consequences imply that the final CO_2_ pressure is controlled by the chemical equilibrium of the carbonation reaction. As the pressure increases, the adsorption capacity increases.

### DFT simulation results

The simplest model that can be considered for the adsorption of CO_2_ by lithium hydroxide is that lithium hydroxide adsorbs carbon dioxide and turns into lithium bicarbonate. Now, we use the Gibbs free energy equation to have a view of how lithium hydroxide reacts with carbon dioxide and forms lithium bicarbonate at room temperature^22^:

19 $$ \Delta G = \Delta H - T\Delta S $$

For this purpose, we calculate the enthalpy and entropy changes for the desired chemical reaction at room temperature, i.e. 298.15 K. Table 6 shows the data related to enthalpy and entropy of the reaction components of carbon dioxide adsorption by lithium hydroxide and the changes in the related thermodynamic parameters are given.

**Table 6:** The data related to enthalpy and entropy of the reaction components of CO_2_ adsorption by lithium hydroxide.

The reaction components	H (kcal/mol)	S (kcal/mol K)	
LiOH	10.78	0.05	
CO_2_	9.11	0.05	
LiHCO_3_	21.44	0.07	

Carbon dioxide adsorption reaction	∆H (kcal/mol)	∆S (kcal/mol.K)	∆G (kcal/mol)
LiOH + CO_2_ → LiHCO_3_	1.55	− 0.03	9.95

**Figure 13:**
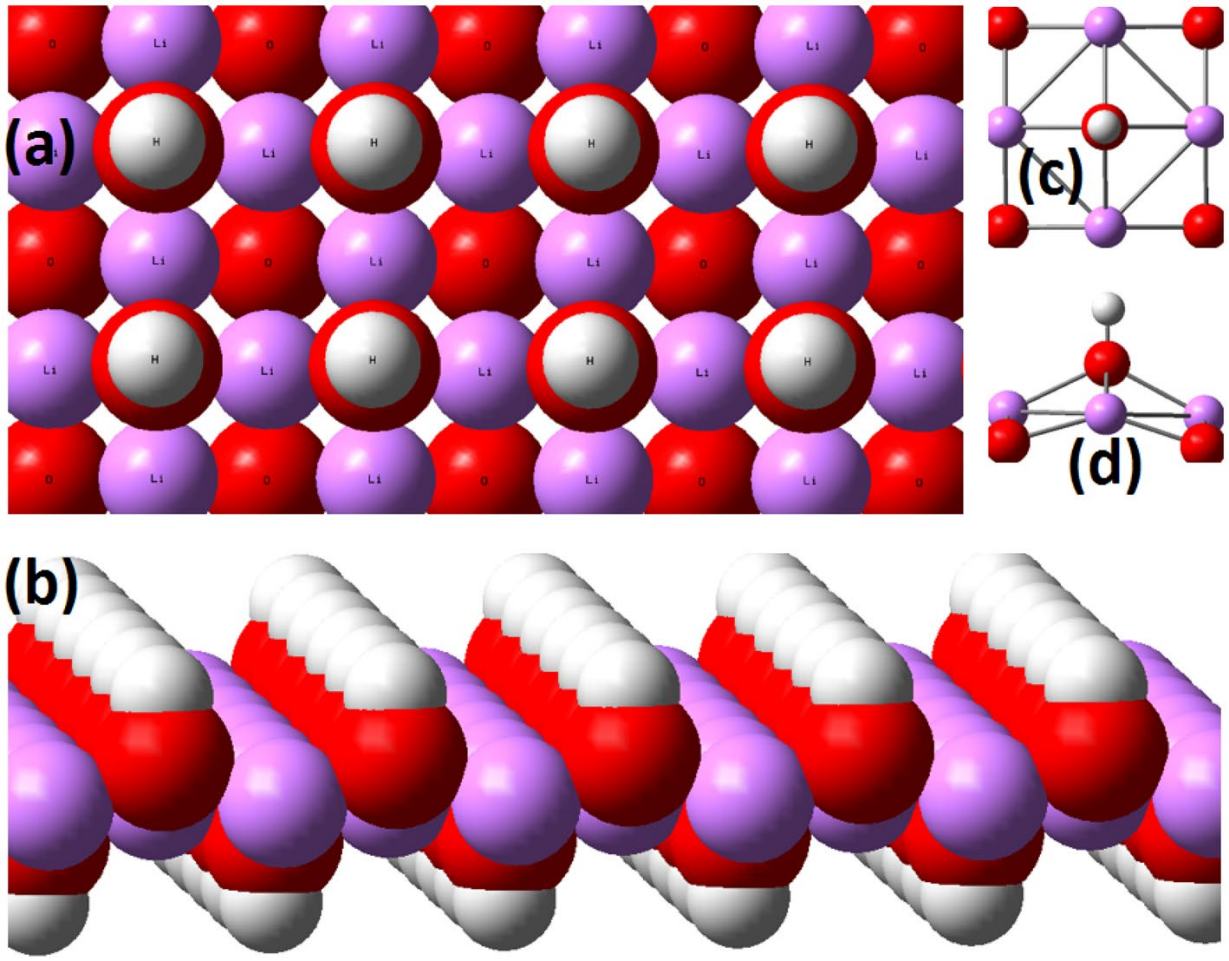
A structure of single layer crystal of lithium hydroxide. (**a**) and (**b**) show the top and side views of the monolayer crystal structure, respectively, and (**c**) and (**d**) show the top and side views of the repeating unit of the lithium hydroxide crystal.

Gibbs free energy changes indicate that the process of chemical adsorption of carbon dioxide by lithium hydroxide cannot be spontaneous at room temperature, for this process to be spontaneous; the ambient temperature needs to be less than 55 K. Now we will check whether there is physical adsorption between lithium hydroxide and carbon dioxide at room temperature or not. We first modeled a single layer of lithium hydroxide crystal structure; you can see its model in Fig. 13.

To estimate the amount of attraction or repulsion between lithium hydroxide and CO_2_, we calculate the binding energy between several atomic clusters of lithium hydroxide and CO_2_ in the optimal state relative to each other. We calculate the size of the desired dependence energy from the following equation^68^.

20 $$ E_{b} = E_{{{\text{CO}}2}} + E_{Cluster} - E_{Combination} $$

**Figure 14:**
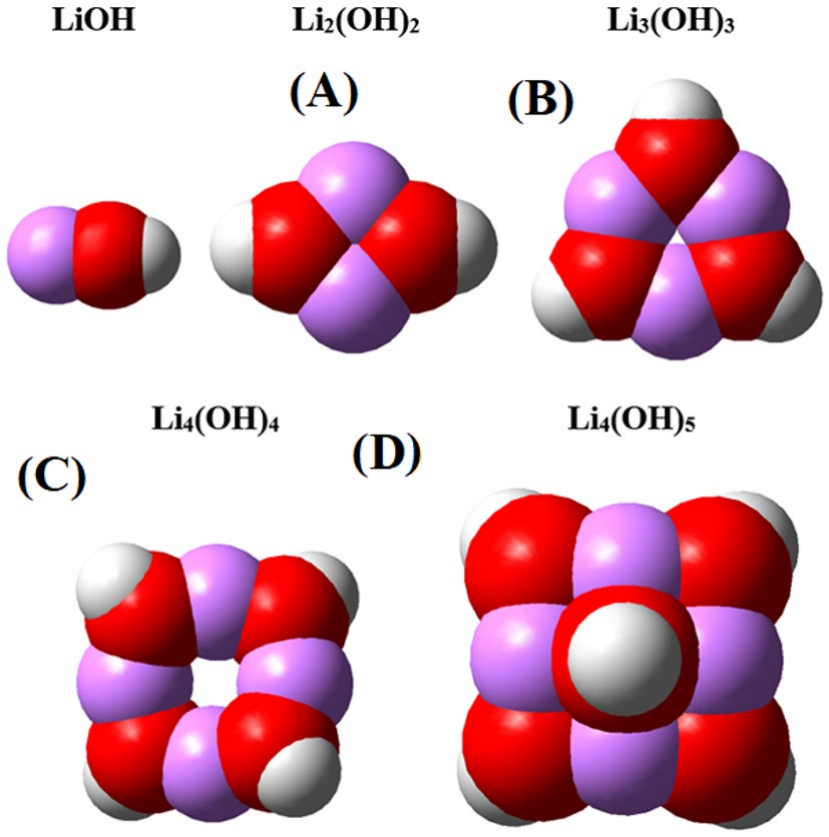
A structure of single layer crystal of lithium hydroxide. Figureures A and B show the top and side views of the monolayer crystal structure, respectively, and Figureures C and D show the top and side views of the repeating unit of the lithium hydroxide crystal.

In Fig. 14, the lithium hydroxide atomic clusters investigated in this research, which are LiOH, Li_2_(OH)_2_, Li_3_(OH)_3_, Li_4_(OH)_4_ and Li_4_(OH)_5_, are shown in the optimal structures obtained by density functional theory calculations. Note that the Li_4_(OH)_5_ cluster is the smallest salt crystal that can be seen repeatedly in the lithium hydroxide crystal structure.

By examining the chemical hardness of the above atomic clusters, we can conclude that the above structures have acceptable chemical stability. The chemical hardness can be obtained for each atomic cluster of lithium hydroxide using the following relationship.

21 $$ \eta = \frac{{E_{LUMO} - E_{HOMO} }}{2} $$

For each of the introduced atomic clusters, the highest occupied molecular orbital (HOMO), the lowest unoccupied molecular orbital (LUMO) and the HOMO–LUMO gap and finally the chemical hardness were obtained (Table S4 in the supplementary).

By observing the chemical hardness of lithium hydroxide atomic clusters, we conclude that these structures are chemically stable. Usually, with the nanonization of materials their chemical reactivity increases; but with the nanonization of lithium hydroxide, it turns from a salt structure into covalent atomic clusters. For the above structures, there are no imaginary vibrations (For example, the IR frequency spectrum for Li_4_(OH)_5_ is shown in Fig. S6 in the supplementary).

Now, after determining the chemical stability of lithium hydroxide atomic clusters, we consider a compound system between the atomic clusters introduced with carbon dioxide, and concluded that there will be physical attraction between lithium hydroxide and carbon dioxide clusters in the optimal state. In Table 7, there is information about the size of the clusters, the optimal distance between the clusters and carbon dioxide, and finally the binding energy between each of the clusters and carbon dioxide in the optimal state.

**Table 7:** Information of the clusters, the optimal distance between the clusters and carbon dioxide, and finally the binding energy between each of the clusters and carbon dioxide in the optimal state.

Clusters	Size (nm)	Distance (Å)	E_b_
LiOH	0.26	1.9	1.08
Li_2_(OH)_2_	0.47	2.0	0.37
Li_3_(OH)_3_	0. 50	2.1	0.26
Li_4_(OH)_4_	0.65	2.1	0.23
Li_4_(OH)_5_	0.66	2.1	0.11

**Figure 15:**
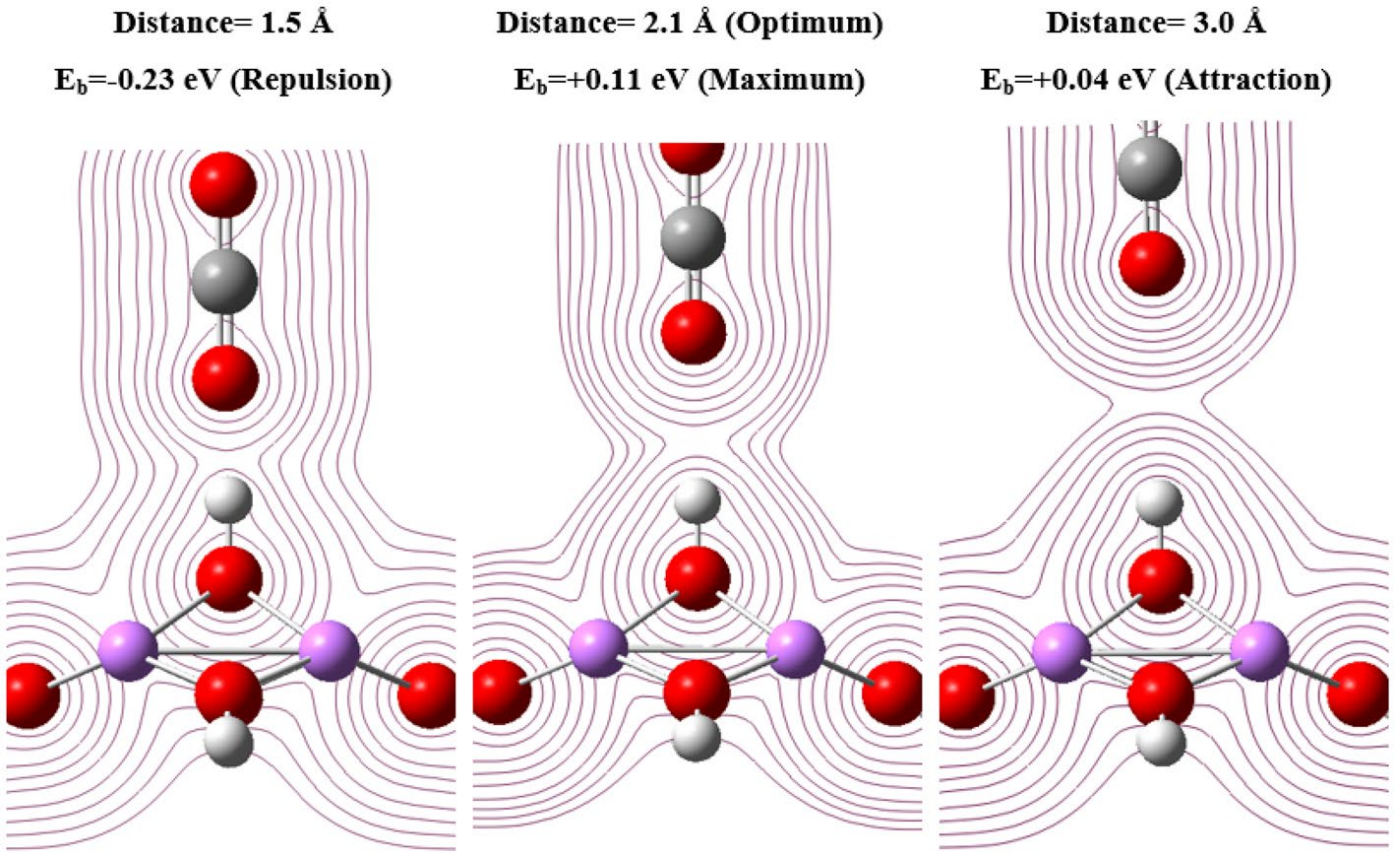
The state of the carbon dioxide molecule and the lithium hydroxide in three states: closer to the optimum, D = 1.5; the optimum state, D = 2.1; and the farthest state from the optimum, i.e. D = 3 Angstrom.

The first result that can be obtained from the data in the above table is that as the clusters get bigger, their optimal distance with the carbon dioxide molecule tends to a limit value, which is around 2.1 angstroms here. This distance is the same value that the carbon dioxide molecule can have in the optimal state with the lithium hydroxide salt crystal. The second point that can be noticed is that the smaller the size of the clusters, the greater its attraction to carbon dioxide, or in other words, the attraction of small clusters is closer to chemical adsorption. And finally, there is a third point about the Li_4_(OH)_5_ cluster, which, as mentioned, is the smallest crystal structure of lithium hydroxide salt. You see the binding energy for this structure is different and lower than the other clusters in the table, almost half of the value of the similar structure i.e. Li_4_(OH)_4_. Because for this cluster, we assumed the optimal position of the carbon dioxide molecule in a position where the carbon dioxide is close to the hydroxide part of the salt. This assumption was different from the situation that existed for other clusters because in those clusters, carbon dioxide approaches the lithium atoms of the cluster from its oxygen side. The reason for this assumption is that in the crystal structure, lithium atoms are placed in the inner part of the salt structure, and the outermost part of the salt crystal is the hydroxide part placed on the lithium atoms (See part b of Fig. 2 in the supplementary file). As mentioned in the table above, the optimal distance of carbon dioxide from lithium hydroxide is about 2.1 angstroms, which means that the maximum binding energy occurs at this distance, and if the distance between carbon dioxide and salt is less than this, it will face electrostatic repulsion. This conclusion is shown schematically in Fig. 15.

**Figure 16:**
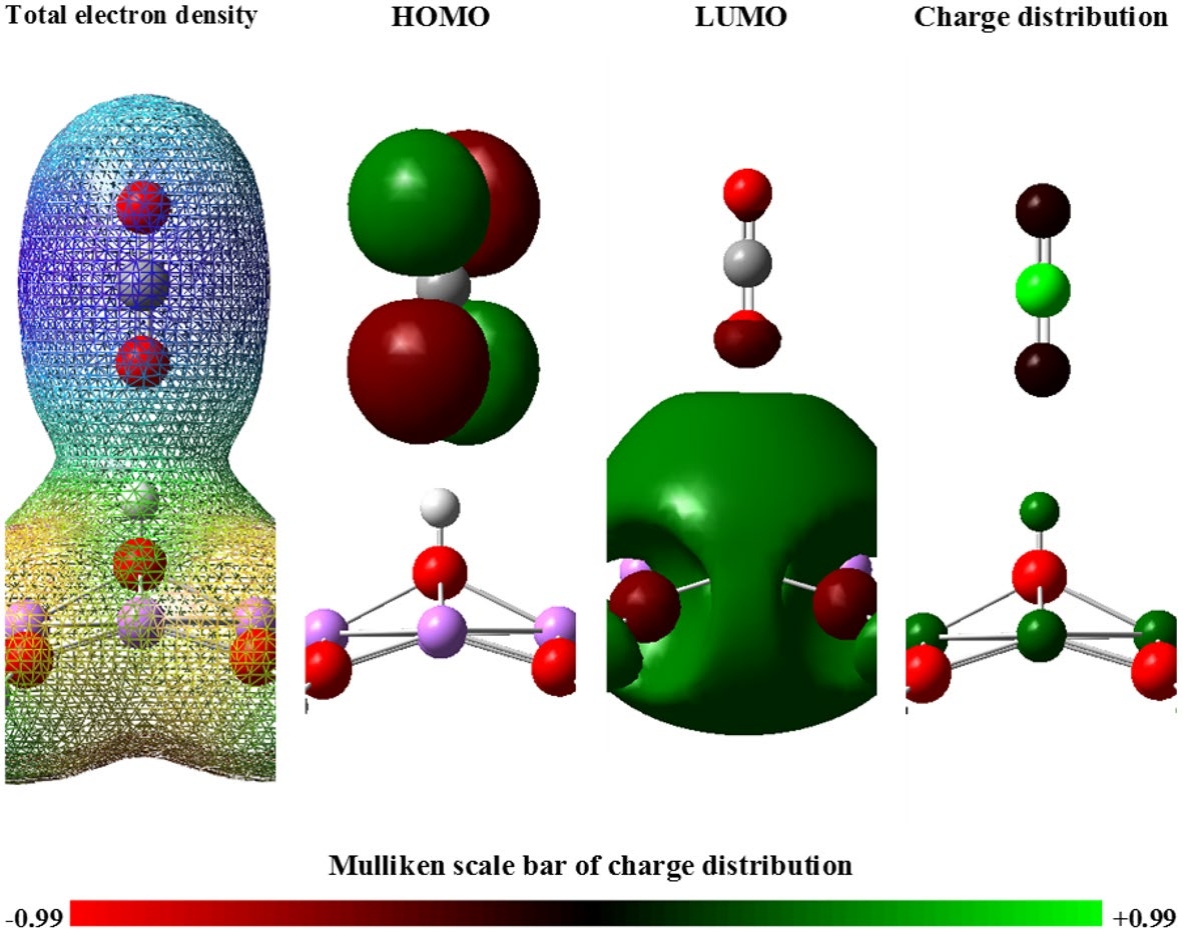
The electron cloud distribution, the position of the HOMO and LUMO orbitals and finally the Mulliken charge distribution for the combination of carbon dioxide-lithium hydroxide.

In fact, the combination of carbon dioxide and lithium hydroxide can form a one-piece system in terms of electron cloud distribution, which will have the lowest possible energy in the optimum state of this electron distribution. In the combination of carbon dioxide-lithium hydroxide, the orbitals of carbon dioxide molecule will play the role of HOMO, and the orbitals of lithium hydroxide will play the role of LUMO of the compound. For this reason, the attraction between carbon dioxide and lithium hydroxide is justified, and their distance should be such that this combined system has the lowest potential energy, in other words, the two compounds are in an optimum position to each other. In Fig. 16, you can see the situation of electron cloud distribution and HOMO and LUMO orbitals in the desired combination.

According to the charge distribution on the Mulliken scale, it is clear that the oxygens in the carbon dioxide molecule act as the negative pole and the hydrogens on the surface of the lithium hydroxide act as the positive pole of the structure, so initially there is an attraction between carbon dioxide and lithium hydroxide. However, as the carbon dioxide molecule approaches the surface of the lithium hydroxide crystal, the repulsive force between the carbon of the molecule and the hydrogen of the surface increases and decreases the attractive force. Then, as the molecule approaches the salt, the interaction force between the two turn into repulsive.

## Conclusion

A remarkable difference was observed in the CO_2_ adsorption behaviors on the LiOH. The effects of various operating parameters, i.e., LiOH particle size, temperature, and pressure, on the CO_2_ adsorption capacity were wisely examined using the RSM method. The CO_2_ uptake rapidly increased, with increasing pressure from 1 to 9 bar, and accordingly, at an equilibrium pressure of 9 bar, the maximum CO_2_ uptake (318.095 mg/g) attained by the LiOH solid which is significantly higher than the amount obtained in the pressure of 1 bar (156.242 mg/g). Hill isotherm model to the closest fit to the experimental data was two-parameter model. The second-order closest model to fit the experimental data was a kinetic one. An increase in temperature led to an increase in the chemical CO_2_ uptake of the solid lithium hydroxide. The values of ΔH° and ΔS° were calculated from the slopes and intercepts of linear regression of Ln k_d_ versus 1/T, based on which ΔH° = − 13,681 j/mol and ΔS° = − 72 j/mol·K were obtained, representing that the exothermic chemical reaction is between carbon dioxide and lithium hydroxide. Also, our DFT simulations show that with the nanonization of lithium hydroxide, are formed stable atomic clusters, and the smaller the dimensions of these clusters, the greater the attraction between them and the carbon dioxide molecule.

## Supplementary Information

Supplementary Information.

## Data Availability

The datasets used and/or analyzed during the current study available from the corresponding author on reasonable request.

## List of symbols

K_L_: Langmuir constant, bar^−1^
k_F_: Freundlich constant, cm^3^/g bar^1/n^
K_d_: Hill constant, cm^3^/g bar^n^
k_f_: Rate constant of pseudo-first order adsorption, min^−1^
k_s_: Rate constant of the pseudo-second order kinetics, g mg^−1^ min^-1^
k_2_: Reaction rate constant of Ritchie second order equation, min^−1^
k_id_: Intraparticle diffusion rate constant, mg g^−1^ min^−1^
M_CO__2_: Molar mass of carbon dioxide, g mol^−1^
m: Mass of adsorbent, g
N: Total number of experiments required
n: Number of variables
n_H_: Hill cooperativity coefficient of the binding interaction
P_i_: Initial pressure, bar
FT-IR: Fourier transform-infrared spectroscopic analysis
LiOH: Lithium hydroxide
P_e_: Equilibrium pressure, bar
q_e_: Equilibrium adsorption capacity, mg/g, cm^3^/g
$q\overline{e}$: Average of *q*_e_, mg/g
q_m_: Maximum CO_2_adsorption capacity, cm^3^/g
q_s_: Hill isotherm maximum uptake saturation, mg/L
q_t_: Amount of adsorbed CO_2_ at time t
R: Universal gas constant, 8.314 J mol^−1^ K^−1^
R^2^: Correlation coefficient
R_L_: Dimensionless constant
T: Temperature of the reactor, K
t: Reaction time, min
V: Volume of the reactor occupied by the CO_2_ gas, ml
W: Grams of adsorbent, g
XRD: X-ray diffraction
CCD: Central composite design

## Greek letters

α: Initial adsorption rate, mg g^−1^ min^−1^
β: Desorption constant, g mg^−1^
λ: D-R constant, mol^2^/J^2^
ω: Polanyi potential (equivalent to RT ln(1/1 + P))
ΔH°: Enthalpy change, kJ mol^−1^
ΔS°: Entropy change, kJ mol^−1^ K^−1^
ΔG°: Gibbs free energy change, kJ mol^−1^
