# Supplementary material for: Lithium hydroxide as a high capacity adsorbent for CO2 capture: experimental, modeling and DFT simulation
Source: Sci Rep. 2023 May 2;13:7150. doi: 10.1038/s41598-023-34360-z (PMC10154391; doi:10.1038/s41598-023-34360-z)
Supplement: Supplementary file 1 — Supplementary Information. [file 41598_2023_34360_MOESM1_ESM.docx]

The number of tables and research results are attached.


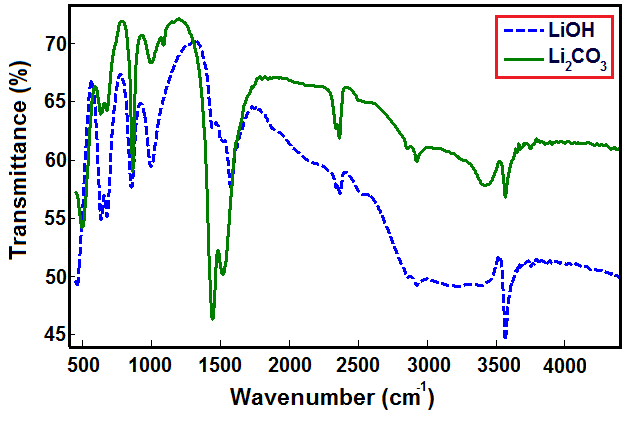


**Fig 1. FTIR spectra of LiOH and Li_2_CO_3_.**


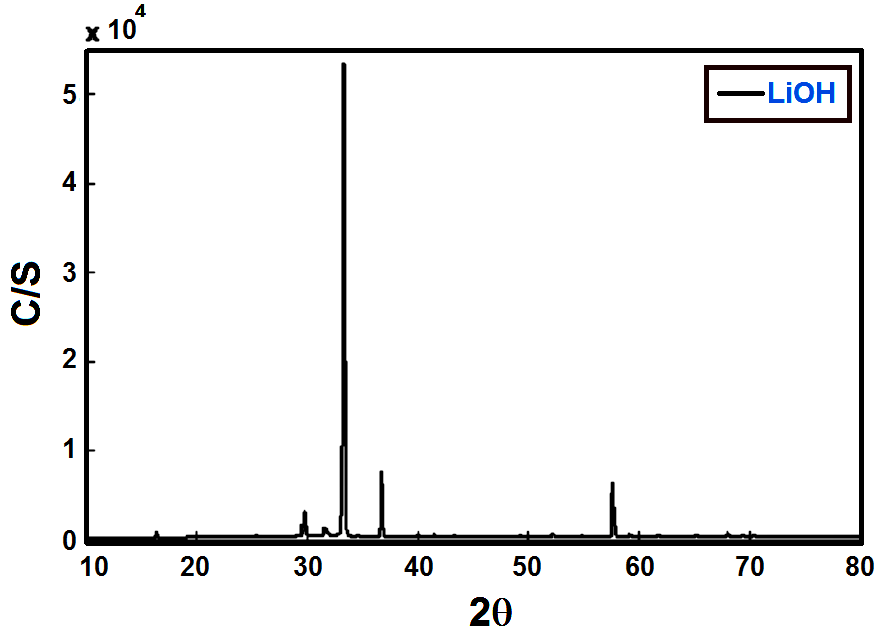


**Fig 2. XRD patterns of LiOH solid**


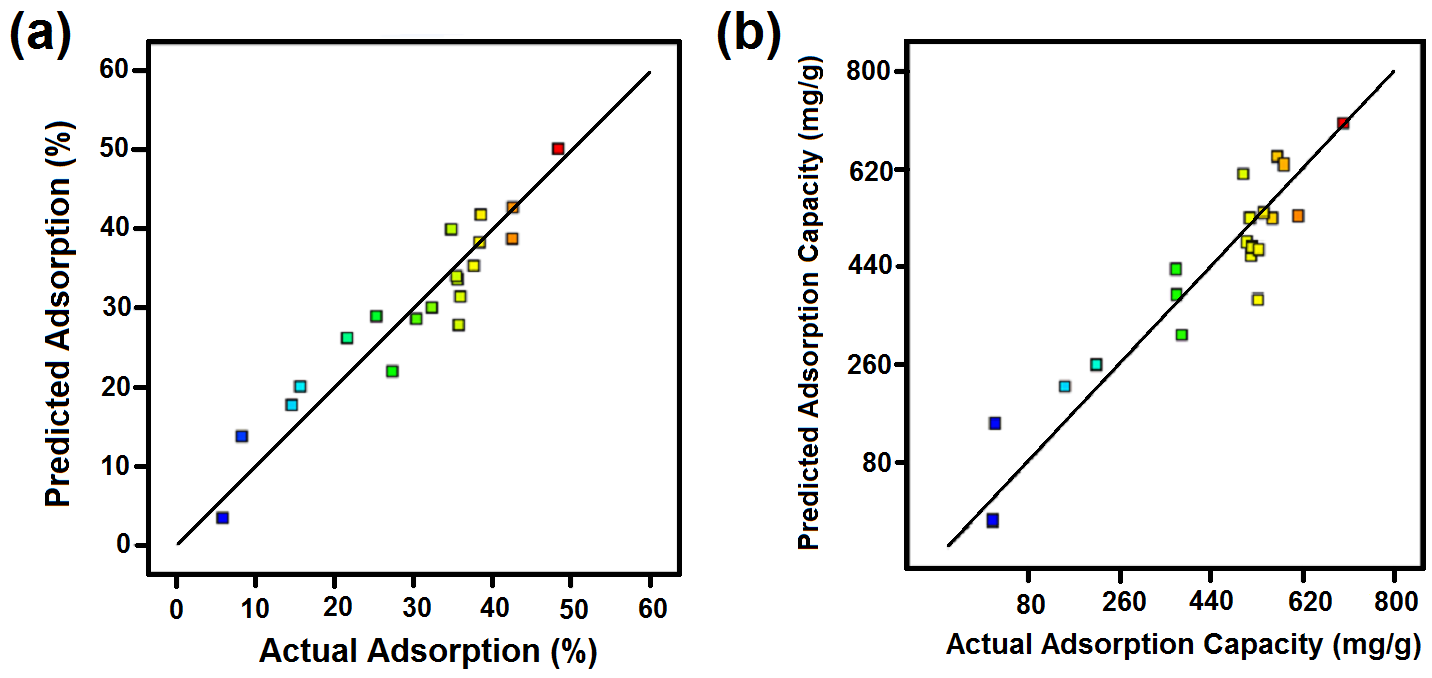


**Fig 3. Predicted vs. experimental values of CO_2_, (A) adsorption percentage, and (B) adsorption capacity.**

**Fig 4. Variation of CO_2_ adsorption percentage with temperature.**


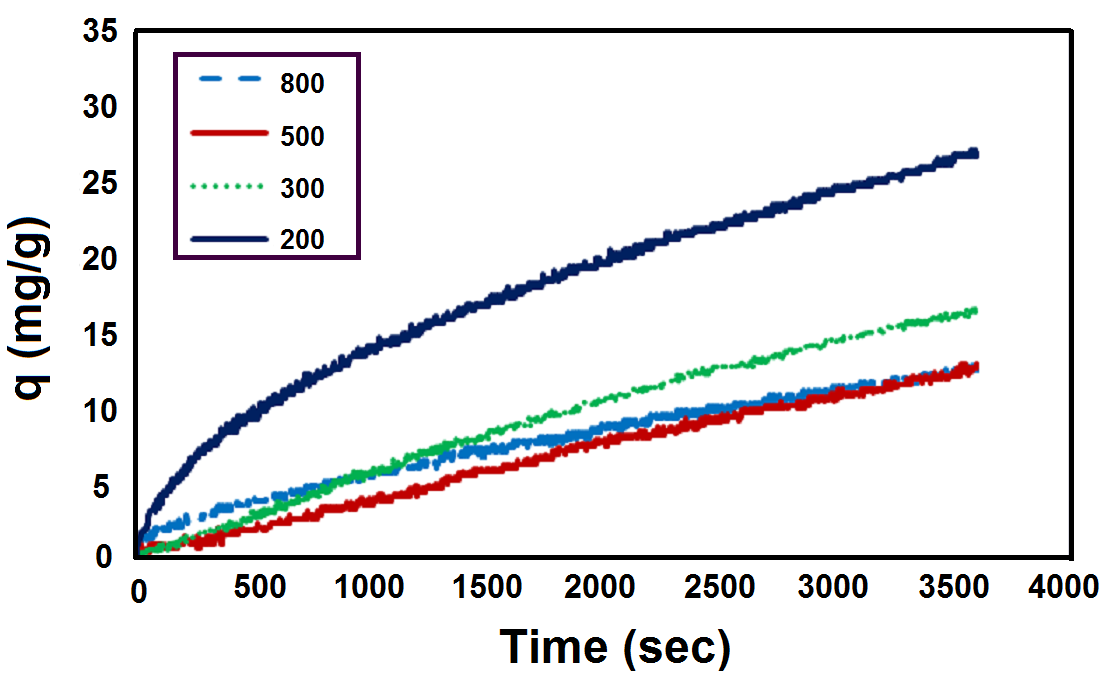


**Fig 5. The effect of particle size on carbon dioxide adsorption capacity (mg/g) at 303 K and pressure of 6 bar**


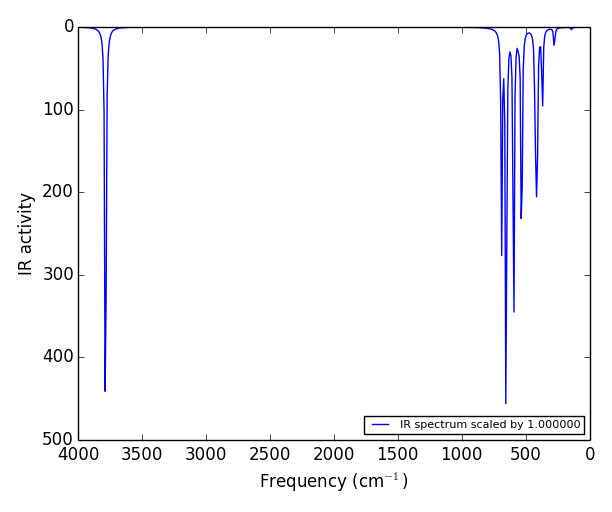


**Fig 6. IR spectrum for the Li_4_(OH)_5_ structure.**

**Table 1. The effects of the operation conditions on CO_2_ capture performance**

| Run | T(C) | P(bar) | Mesh size(µm) | q(mg/g) | %Ad. |
| --- | --- | --- | --- | --- | --- |
| 1 | 30 | 1 | 800 | 10.79 | 5.80 |
| 2 | 30 | 3 | 500 | 382.27 | 27.28 |
| 3 | 90 | 9 | 200 | 700.45 | 48.33 |
| 4 | 50 | 6 | 200 | 543.33 | 35.73 |
| 5 | 30 | 1 | 200 | 15.78 | 8.20 |
| 6 | 90 | 9 | 800 | 611.52 | 42.52 |
| 7 | 70 | 3 | 200 | 560.52 | 37.61 |
| 8 | 50 | 3 | 500 | 372.27 | 21.57 |
| 9 | 70 | 6 | 500 | 503.45 | 34.78 |
| 10 | 50 | 1 | 200 | 214.57 | 15.62 |
| 11 | 30 | 6 | 200 | 518.84 | 30.34 |
| 12 | 60 | 3 | 300 | 533.72 | 35.92 |
| 13 | 70 | 9 | 800 | 510.42 | 35.57 |
| 14 | 90 | 1 | 200 | 516.73 | 38.36 |
| 15 | 70 | 1 | 300 | 532.57 | 35.72 |
| 16 | 70 | 6 | 300 | 570.33 | 38.53 |
| 17 | 30 | 9 | 200 | 520.79 | 32.31 |
| 18 | 70 | 9 | 300 | 583.36 | 42.56 |
| 19 | 30 | 9 | 800 | 371.25 | 25.27 |
| 20 | 90 | 1 | 800 | 152.65 | 14.56 |

Table 2. ANOVA results for RSM-CCD model of q_e_ response.

| **Analysis of variance** (**q_e_**) | | | | | |
| --- | --- | --- | --- | --- | --- |
| **Source** | **Sum of Squares** | **df** | **Mean Square** | **F-Value** | **p-value**  **Prob > F** |
| Model | 6.200e5 | 9 | 6.200e5 | 7.25 | 0.0023 |
| A-Temperature | 1.333e5 | 1 | 1.333e5 | 14.04 | 0.0038 |
| B-Pressure | 3.117e5 | 1 | 3.117e5 | 32.83 | 0.0002 |
| C-Mesh size | 83314.11 | 1 | 83314.11 | 8.77 | 0.0142 |
| AB | 11915.87 | 1 | 11915.87 | 1.26 | 0.2888 |
| AC | 9831.45 | 1 | 9831.45 | 1.04 | 0.3329 |
| BC | 11701.26 | 1 | 11701.26 | 1.23 | 0.2929 |
| A^2^ | 854.41 | 1 | 854.41 | 0.09 | 0.7703 |
| B^2^ | 26575.98 | 1 | 26575.98 | 2.80 | 0.1253 |
| C^2^ | 7796.25 | 1 | 7796.25 | 0.82 | 0.3862 |
| Residual | 9494.05 | 10 | 9494.0 | - | - |
| Cor Total | 7.149e5 | 19 |  | - | - |

**Table 3. Adsorption kinetic models**

| Eq. Nu. | Nonlinear Form | Models |
| --- | --- | --- |
| (13) | **$q_{t}=q_{e}.\left(1-\exp\left(-k_{1}.t\right)\right)$** | First order |
| (14) | **$q_{t}=k_{2}.q_{e}^{2}\frac{t}{\left(1+k_{2}.q_{e}.t\right)}$** | Second order |
| (15) | **$q_{t}= \beta.\log\left(\alpha.\beta\right)+\beta.\text{log}\left(t\right)$** | Elovich equation |
| (16) | **$q_{t}=k_{id}.t^{0.5}$** | Rate controlling [39] |

**Table 4. Information of the clusters, the optimal distance between the clusters and carbon dioxide, and finally the binding energy between each of the clusters and carbon dioxide in the optimal state**

| Clusters | HOMO (eV) | LUMO (eV) | E_Gap_ (eV) | η |
| --- | --- | --- | --- | --- |
| LiOH | -5.72 | -1.27 | 4.45 | 2.22 |
| Li_2_(OH)_2_ | -6.08 | -0.51 | 5.57 | 2.78 |
| Li_3_(OH)_3_ | -6.57 | -0.48 | 6.09 | 3.05 |
| Li_4_(OH)_4_ | -6.67 | -0.53 | 6.14 | 3.07 |
| Li_4_(OH)_5_ | -7.67 | -0.35 | 7.32 | 3.66 |
